# Abscisic Acid Rescues Behavior in Adult Female Mice in Attention Deficit Disorder with Hyperactivity Model of Dopamine Depletion by Regulating Microglia and Increasing Vesicular GABA Transporter Expression

**DOI:** 10.1007/s11481-025-10186-6

**Published:** 2025-04-16

**Authors:** Maria Meseguer-Beltrán, Sandra Sánchez-Sarasúa, Nóra Kerekes, Marc Landry, Matías Real-López, Ana María Sánchez-Pérez

**Affiliations:** 1 Department of Medicine, Universitat Jaume I, Castellón, Spain; 2 Institute of Neurodegenerative Diseases, CNRS, University of Bordeaux. UMR 5293, Bordeaux, France; 3 Department of Health Sciences, University West, 46186 Trollhättan, Sweden; 4 Serious Mental Disorder Program in Childhood and Adolescence, Provincial Hospital Consortium of Castellón, Castellón, Spain

**Keywords:** 6-OHDA lesion, VGAT, VGluT1, E/I ratio, IL-1β, Arg1, Anterior cingulate cortex, posterior insular cortez, Hippocampus

## Abstract

Attention-deficit/hyperactivity disorder (ADHD) is a neurodevelopmental syndrome typically diagnosed in childhood that may persist into adulthood. Its etiology encompasses both genetic and environmental factors, with genetic studies indicating catecholamine dysfunction and epidemiological evidence emphasizing neuroinflammation as a potential trigger. To investigate the roles of inflammation and development processes in ADHD, we conducted a longitudinal behavioral study using female Swiss mice with a dopamine deficit model. We explored the impact of neonatal dopaminergic lesions, treatment with abscisic acid (ABA)—an anti-inflammatory hormone—and developmental changes by comparing behavioral patterns in juvenile and adult mice. Postmortem analyses assessed neuroinflammation through microglial morphology, NLRP3, cytokine expression, and the excitatory/inhibitory (E/I) ratio in specific brain regions. Neonatal dopaminergic lesions induced hyperactivity and hypersensitivity in juvenile mice that persisted into adulthood. In adults, increased social interaction and memory impairment were observed in lesioned mice. Brain development mitigated impulsivity, while ABA treatment reduced locomotor activity, downregulated pain sensitivity, and influenced social interaction, although it did not completely resolve cognitive deficits in lesioned adult mice. In brain regions such as the anterior cingulate cortex (ACC), posterior insular cortex (pIC), and hippocampus, lesions significantly altered microglial morphology. In the ACC, lesions increased IL-1β and TNFα levels, decreased Arg1 mRNA levels, and disrupted the E/I balance. Importantly, ABA treatment restored microglial morphology, normalized IL-1β and Arg1 expression and upregulated vGAT levels. This study demonstrates that dopamine deficits lead to microglia alterations and E/I imbalance, contributing to ADHD symptoms. While some symptoms improve with brain development, targeting microglial health in specific brain regions emerges as a promising therapeutic approach for managing ADHD.

**Graphical Abstract:**

**Effect in adult females of neonatal dopamine depletion and ABA treatment.** Brain Neonatal 6-OHDA dopaminergic lesion induces behavioral hyperactivity, impulsivity, hypersensitivity and increased social interaction in P21 and P90 females, and memory impairment in P90. Two-months of ABA treatment improved hyperactivity, anxiety, hypersensitivity, and alterations in social interaction, but not cognitive impairment. In the ACC of young adult mice (P60) dopamine deficiency induced mRNA alteration (as indicated); and E/I imbalance. ABA treatment restored microglia morphology, IL-1β expression, and increased vGAT levels. Black arrows indicate changes at P90 compared to P21 of the same condition; blue arrows indicate changes at P21, compared to SHAM.

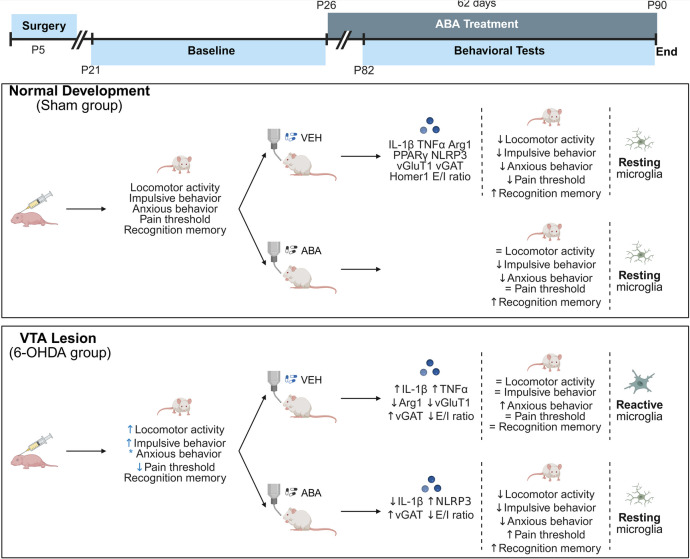

**Supplementary Information:**

The online version contains supplementary material available at 10.1007/s11481-025-10186-6.

## Introduction

Attention-deficit/hyperactivity disorder (ADHD) is a neurodevelopmental syndrome affecting 5 to 10% of children and persisting into adulthood, with a global adult prevalence of up to 2.5% worldwide (Faraone et al. 2015). The disorder profoundly impacts patients' functioning and quality of life (Agarwal et al. 2012; Pawaskar et al. 2020; Velő et al. 2021), significantly increasing the risk of educational and occupational challenges. Individuals with untreated ADHD face heightened risks of social disability, criminal behavior, and addiction (Pratt et al. 2002; Mordre et al. 2011; Engelhardt et al. 2019).

ADHD is characterized by core symptoms of hyperactivity, impulsivity, and inattention, which do not always manifest simultaneously. Patients may present predominantly inattentive or predominantly hyperactive-impulsive symptoms. The disorder frequently coexists with other psychiatric conditions, including anxiety (Guttmann-Steinmetz et al. 2010), depression (Avni et al. 2018), and somatic complaints like hypersensitivity to mechanical or thermal stimuli (Kerekes et al. 2021). This complex clinical presentation underscores the multifaceted etiology of ADHD.

The disorder's onset is associated with catecholamine dysfunction, leading to medications that enhance dopamine (DA) and norepinephrine signaling (Solanto 2002). However, chronic use of these drugs presents significant challenges, including potential tolerance, dependence, and addiction risks, particularly in vulnerable populations (Winhusen et al. 2011). Moreover, adverse effects such as cardiovascular complications and depression have been documented (Sciberras et al. 2022).

Neurobiologically, ADHD is closely linked to alterations in the anterior cingulate cortex (ACC), a critical neural hub that receives sensory information and dopamine input (Bush et al. 2005; Tripp and Wickens 2009). The ACC plays a crucial role in modulating behavioral traits associated with ADHD symptoms, including inattention, impulsivity, and hyperactivity (Newman and McGaughy 2011; Golchert et al. 2017). ACC receives sensory information via the thalamus and DA input from the ventral tegmental area (VTA) (López-Avila et al. 2004). Additionally, it influences pain perception and anxious behavior through neural connections with other brain regions, such as the posterior insula (pIC) (Gamal-Eltrabily et al. 2021) and the amygdala (Klumpp et al. 2017; López-Cruz et al. 2017). ACC hyperexcitability is therefore associated with increased pain sensitivity (Bai et al. 2019) and anxiety (Twillman 2007). The ACC functions as a central hub controlling pain and pain-related emotions (Shackman et al. 2011; Bliss et al. 2016).

Emerging research suggests that early developmental inflammation represents a significant risk factor for ADHD (Anand et al. 2017; Leffa et al. 2019; Dunn et al. 2019; Chen et al. 2022). Thus, microbial dysbiosis (Gkougka et al. 2022) and maternal autoimmune reactions (Ellul et al. 2022) have been implicated in the disorder´s incidence and associated conditions (Saccaro et al. 2021).

Microglia, crucial for neural network maintenance, play a pivotal role in this process (Paolicelli et al. 1979; Schafer et al. 2012) by eliminating synapses and releasing neurotrophic factors. During neuroinflammation, microglia-dependent synaptic pruning is disrupted, leading to an imbalance in the excitatory/inhibitory (E/I) ratio and subsequent hyperexcitability (Isbrandt 2017). This E/I imbalance underlies cognitive impairment and altered social and emotional behaviors (Gatto and Broadie 2010; Sohal and Rubenstein 2019), contributing to the etiology of neurological, mental, and developmental disorders (Miyanishi et al. 2021).

Activated microglia release proinflammatory cytokines, such as Interleukin 1β and 18(I L-1β, IL-18) via nucleotide oligomerization domain (NOD) -like receptor subtype 3 (NLRP3) inflammasome signaling (Wang et al. 2007; Clark and Malcangio 2014; Liu et al. 2022), as well as tumor necrosis factor-α (TNF-α) (Qin et al. 2007), reactive oxygen species, glutamate, and other biologically active substances. The microglial inflammatory response involves a complex transition from pro-inflammatory (M1) to anti-inflammatory (M2) states, regulated by specific enzymatic processes. Typically, Arginase 1 (Arg1), which competes with iNOS and reduces nitric oxide production (Cherry et al. 2014; Bou Sader Nehme et al. 2024), is characteristic of the M2 status.

We previously observed microglial alterations in the anterior cingulate cortex (ACC) and posterior insular cortex (pIC) in both female and male mice from a validated ADHD model, the neonatal 6-hydroxydopamine (6-OHDA) lesion model (Bouchatta et al. 2018).

These alterations were reversed by one month of abscisic acid (ABA) treatment, which alleviated pain sensitivity in female mice and hyperactivity in males (Meseguer-Beltrán et al. 2023).

ABA is an evolutionarily conserved hormone (Le Page-Degivry et al. 1986), also synthesized in mammalian cells and with a wide applications disease due to its anti-inflammatory effects (for review see Gharib et al. 2024). For instance, through PPARγ signaling, ABA has been shown to inhibit NLRP3 inflammasome activation and reduce oxidative stress (Zhao et al. 2021).

Based on these findings, we hypothesized that long-term ABA treatment in the dopamine deficit model of ADHD would alleviate symptoms by modulating microglial activity, the NLRP3 inflammasome, and the E/I balance in specific brain regions. Additionally, since brain maturation may influence ADHD symptoms, we compared behavior at postnatal day 21 (P21) and P90. To account for potential developmental influences on ADHD symptoms, we comparatively analyzed behavioral characteristics at two distinct developmental stages: postnatal day 21 (P21) and postnatal day 90 (P90). In mice, these ages approximately correspond to human infants and young adults, respectively (Semple et al. 2013).

## Methods and Materials

**Fig. 1:**
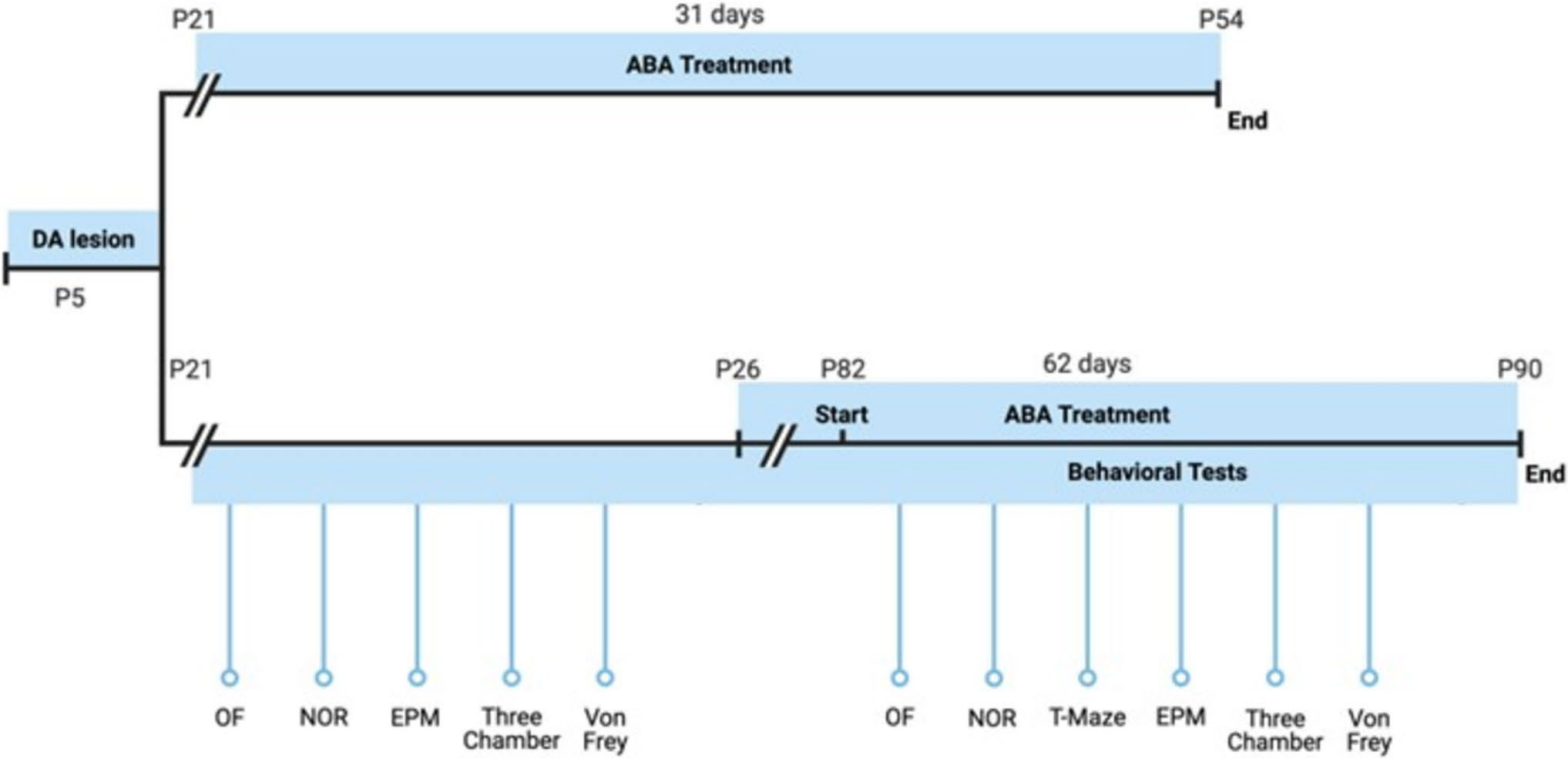
Experiment design timeline. Dopamine lesion (neonatal 6-OHDA injection into lateral ventricle AP − 2 mm, ML ± 0.6 mm, DV − 1.3 mm from Bregma at P5). Baseline started at weaning on P21 until P26. ABA or vehicle administration started on P21 for one month and P26 for two months. Behavioral tests were carried out for one week before terminating the experiment

The time course of experiments is described in Fig. 1.

### Animals and Surgical Procedures

Ninety-three Swiss female mice (Janvier Labs, Saint-Berthevin, France) were housed at the animal facility of the University Jaume I. All procedures adhered to European Community guidelines for the protection of animals used for scientific purposes and were approved by the Ethics Committee of the University Jaume I (scientific procedure 2020/VSC/PEA/0099). The animals were kept on a 12-hour light/dark cycle and provided with food and water ad libitum. Pups were housed with their mothers in a controlled environment at a constant temperature (24ºC ± 2). After weaning, the animals were housed in groups of 2–4 to minimize isolation-induced stress.

The ninety-three female mice were randomly assigned to one of two groups: a sham group (group that underwent surgery with vehicle inoculation); and lesion group. At postnatal day 5 (P5), pups of the lesion group received an injection of 6-hydroxydopamine (6-OHDA) (Sigma-Aldrich, France); and pups of the sham group received vehicle (0.1% ascorbic acid in water) into one of the lateral ventricles, as previously described (Bouchatta et al. 2018).

Thirty minutes before surgery, the mice were pretreated with desipramine hydrochloride (20 mg/kg subcutaneously; Sigma-Aldrich, France), an inhibitor of noradrenergic nerves. Anesthesia was induced with 1% isoflurane and maintained with 0.3% isoflurane during the surgery. A solution containing 25 μg of 6-OHDA dissolved in 3 μL of 0.1% ascorbic acid, or vehicle alone (0.1% ascorbic acid), was infused into one of the lateral ventricles using stereotaxic coordinates (AP −2 mm, ML ±0.6 mm, DV −1.3 mm from Bregma) (Paxinos & Franklin).

After weaning at postnatal day 21 (P21), baseline measurements were taken between P21 and P26. From P26 to P90, mice were randomly assigned to receive either ABA (20 mg/L) or vehicle (0.0008% NaOH) in their drinking water. Animal welfare was closely monitored throughout the study in accordance with the guidelines set by the Ethics Committee.

### Behavioral Procedures

For all behavioral paradigms, mice were acclimated to the testing room for 30 min before each test. The tests were conducted during the day under dim lighting. All apparatuses were cleaned with a 30% ethanol solution between trials and between animals. The mice performed the behavioral paradigms as previously described (Meseguer-Beltrán et al. 2023). Except for Von Frey test, all behavioral parameters studied were analyzed using a video-tracking system (ANY-maze, Stoelting Europe, Dublin, Ireland).

*Open Field*: Spontaneous locomotor activity and anxiety were assessed using the open field test at P21 and P82. Mice were placed in the open field cage facing one of the walls and allowed to explore the arena freely for 10 min. The distance traveled (cm), speed (cm/s), time spent in the center (sec), and latency to cross the center quadrants with all four legs (sec) were recorded.

*Novel Object Recognition* (NOR): Recognition memory was evaluated with the NOR test at P22 and P83. Mice were first allowed to explore two identical objects for 10 minutes (familiarization phase). After 30 minutes, they were returned to the arena and given 10 minutes to explore one familiar object and one novel object (test phase). The first 3 minutes were analyzed, and data were expressed as the discrimination index (DI): ((Time exploring the novel object - Time exploring the familiar object)/Total exploration time). A discrimination index of 0 indicates impaired novelty recognition memory, where the mice explore both objects equally.

*T-maze:* Spatial memory was assessed using the T-maze test at P84. Mice were placed in the starting arm and allowed to explore two of the three arms for 5 minutes (familiarization phase). After a 30-minute intertrial interval in their home cage, mice were returned to the starting position with access to all three arms for 5 minutes (test phase). The previously closed arm was considered the “novel” arm, and the other arm was the “familiar” arm. Data were expressed as the discrimination index (DI): ((Entries into the novel arm − Entries into the familiar arm)/Total entries).

*Elevated Plus Maze* (EPM): Impulsivity was assessed with the EPM test at P23 and P85. Mice were placed in the center of the maze and allowed to explore freely for 10 minutes. Data were expressed as the percentage of time spent exploring the open arms compared to the total time exploring (time spend in closed + open arms but excluding the time in the center) areas delimited in ANY-MAZE software.

*Three Chamber*: Social interaction was assessed using the three-chamber test at P24 and P86. Mice were placed in the central chamber and allowed to explore all chambers freely for 10 minutes (habituation phase). Immediately after, the test phase (10 minutes) was conducted, during which the mouse could explore either an object or a conspecific mouse confined behind a fence in the lateral chambers. The location of the conspecific mouse was balanced, and different conspecifics were used to ensure consistency. Data were expressed as the percentage of time spent exploring the conspecific mouse relative to the total exploration time. Climbing or running around the chamber was not considered exploration.

*Von Frey*: The nociceptive response to a mechanical stimulus was assessed using the von Frey test at P25 and P87. Mice were habituated for 30 minutes in individual cages with a mesh floor. The plantar surface of the hind paws was stimulated with calibrated von Frey filaments of varying force to determine the withdrawal threshold. Three to five measurements were taken for each hind paw, with a 30-second interval between each. The mechanical pain threshold was determined by the filament force (grams) that elicited a paw withdrawal response.

### Immunofluorescence Procedure

Immunofluorescence was performed as described (Sánchez-Sarasúa et al. 2021). Briefly, mice were anesthetized and perfused with saline (0.9% NaCl) followed by fixative (4% paraformaldehyde in 0.1M PB, pH 7.4). After perfusion, the brains were removed, postfixed overnight, and cryoprotected in 30% sucrose in 0.01M PBS pH 7.4 for 3 days. The brains were cut in the rostro caudal direction (40μm) using a sliding microtome Leica SM2010R (Leica Microsystems, Heidelberg, Germany). Primary antibodies mouse anti-Tyrosine Hydroxylase (catalog number MAB318, Sigma-Aldrich, France; 1:5000), rabbit anti-Iba1 (catalog number 019–19741, FUJIFILM Wako Chemicals Europe GmbH, Deutschland; 1:1000), rabbit anti-vGluT1 (catalog number 135–302, Synaptic Systems, Germany; 1:2000), rabbit anti-vGAT (catalog number, 131-002, Synaptic Systems, Germany; 1:1000), mouse anti-Neurofilament-L (catalog number 171-011, Synaptic Systems, Germany; 1:2000), mouse anti-MAP2 (catalog number 13–1500, Invitrogen, Waltham, United States; 1:2000), rabbit anti-Homer1 (catalog number 160-002, Synaptic Systems, Germany; 1:2000) and rabbit anti-NLRP3 (catalog number MA5-32255**,** Thermo Scientific, Rockford, IL, USA; 1:300) were incubated over-night. Next, sections were rinsed and incubated for 2h at RT with donkey anti-mouse Cy3 (catalog number 715-165-150) or donkey anti-rabbit Alexa 488 (catalog number 711-165-152) secondary antibodies (Jackson Immunoresearch, Suffolk, UK). Finally, sections were mounted on slides and covered using Fluoromount-G mounting medium (Invitrogen, California, USA).

### Imaging and Analysis

Fluorescence images were taken with a confocal scan unit with a module TCS SP8 equipped with argon and helio-neon laser beams attached to a Leica DMi8 inverted microscope (Leica Microsystems). Excitation and emission wavelengths for Cy3 were 433 and 560–618nm respectively; Alexa488 labeled excitation wavelength was 488nm and its emission at 510–570nm. For the quantification of Tyrosine Hydroxylase labeling, we used a 10x lens. Image J software was used to count Tyrosine Hydroxylase labeling. Data were expressed as the percentage of Tyrosine Hydroxylase labeling with respect to sham group. For the quantification of Iba1 labeling, we used 20x lens. The custom-designed Image J software macro called “MACROglia” (publicly available at the Github website: https://github.com/SandraSSB/MACROglia_cell-morphology-analysis) combined with FracLac plugin (Karperien) was used to analyze the microglia morphology in sections from sham and 6-OHDA groups as previously described (Espinosa-Fernández et al. 2019). The microglia morphological parameters that were analyzed were (I) fractal dimension (D), this parameter evaluates cellular branching complexity; (II) cell area, meaning the total number of pixels corresponding to the area occupied by the cell, soma, and branches; and (III) cell perimeter, based on the single outline cell shape. For the quantification of double staining vGluT1/NF-L, vGAT/NF-L and Homer1/MAP2, we used 63x lens. Image J software was used to count the number of vGluT1, vGAT and Homer1 points (minimum 3 pixels were considered) in one NF-L or MAP2-positive fiber in 20–25 Z-plane sections from sham and 6-OHDA groups. Ten different axons per animal were analyzed by a researcher blind to the condition. Data is calculated as the number of vGluT1, vGAT and Homer1 positive signals on the NF-L or MAP2 fiber, normalized to the area of the fiber. For the quantification of NLRP3 labeling we used 63x lens. Data were expressed as the mean of gray values per area.

### RNA Extraction and Real-Time Quantitative Polymerase Chain Reaction (RT-qPCR)

Total RNA was extracted from the ACC (*n* = 51) and homogenized in 350μL of lysis buffer according to the PureLink™ RNA Mini Kit (Thermo Scientific, product no. 12183018A, Rockford, IL, USA). Genomic DNA was removed using a spin-column process during the RNA extraction. In addition, DNAse I treatment (Thermo Scientific, Rockford, IL, USA) was performed to ensure the complete removal of genomic DNA. RNA samples were eluted in 20μL of nuclease-free water and reverse transcribed to cDNA using a High-Capacity cDNA Reverse Transcription Kit (Thermo Scientific, product no. 4368814, Rockford, IL, USA) following the manufacturer’s instructions. RT-qPCR reactions were carried out using Maxima SYBR Green/ROX qPCR MM (Thermo Scientific, product no. K0221, Rockford, IL, USA) in an Applied Biosystems StepOne Plus™ Real-Time PCR System (Foster City, CA, USA). The list of primers is presented in Supplementary Table. At the end of each PCR reaction, a melting curve stage was performed to confirm that only one PCR product was amplified in these reactions. The relative gene expression to SEM was calculated by using the 2^−ΔΔCt^ method for each reaction and by using the housekeeping gene GAPDH as internal control.

### Statistical Analysis

**Fig. 2:**
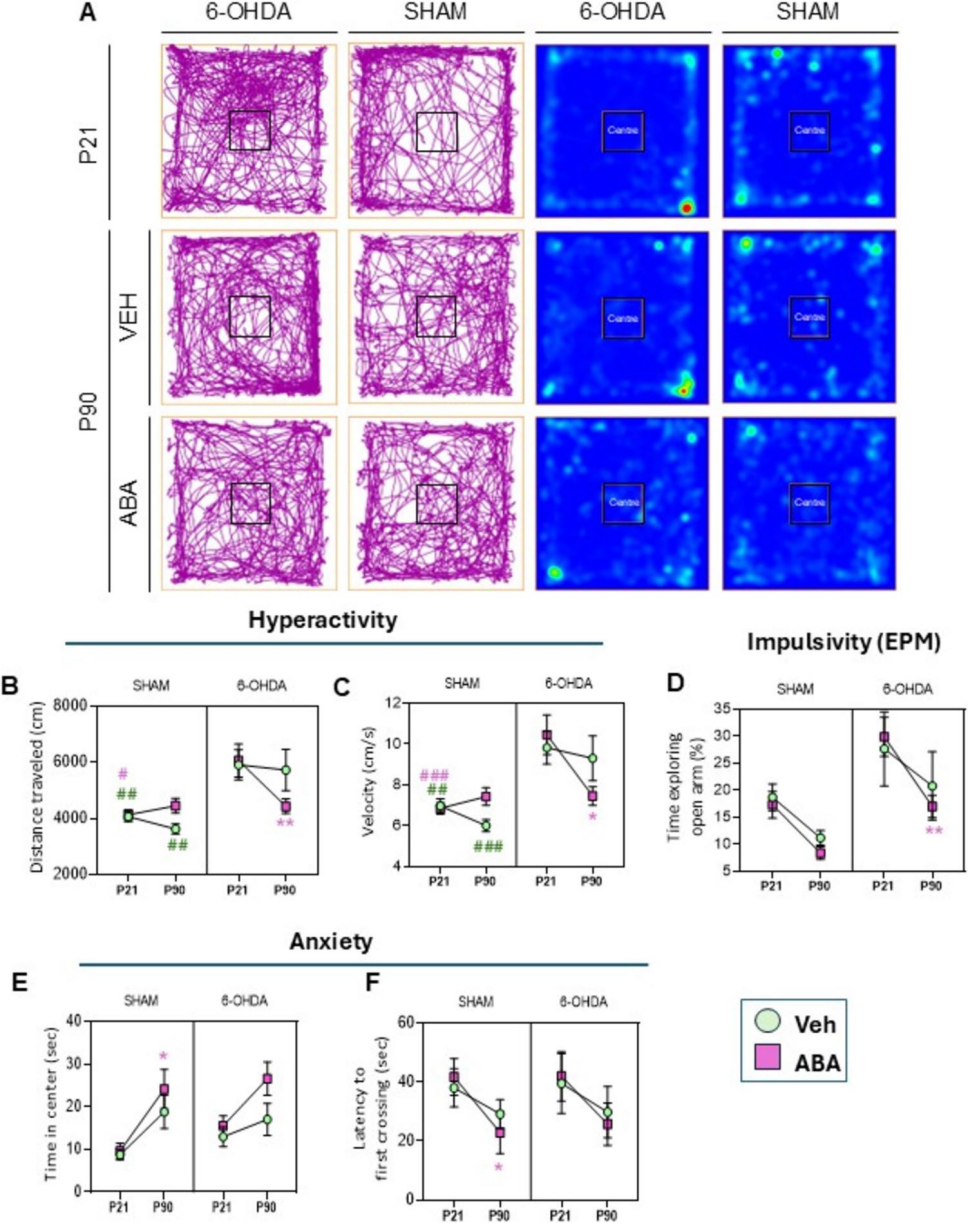
Neonatal 6-OHDA lesion induces hyperactivity, and risk-taking behavior in young and adult mice, and anxiety in adults lesioned mice. ABA administration normalizes locomotor hyperactivity and alleviates normal development-induced anxiety in adults. (**A**) Representative tracking plot and heat map showing alteration in locomotion and exploratory behavior in open field test. (**B**) Distance travelled (cm) and (**C**) speed (cm/s) in the arena. (**D**) Ratio of time exploring open arms/ time in open plus closed arms, in the EPM. (**E**) Time (sec) and (**F**) latency to the first cross into the center (sec) of the arena. Data are presented as mean ± SEM (*n* = 8–11 per condition) and analyzed by Three way-ANOVA with repeated measures, followed by post hoc multiple comparisons test

**Fig. 3:**
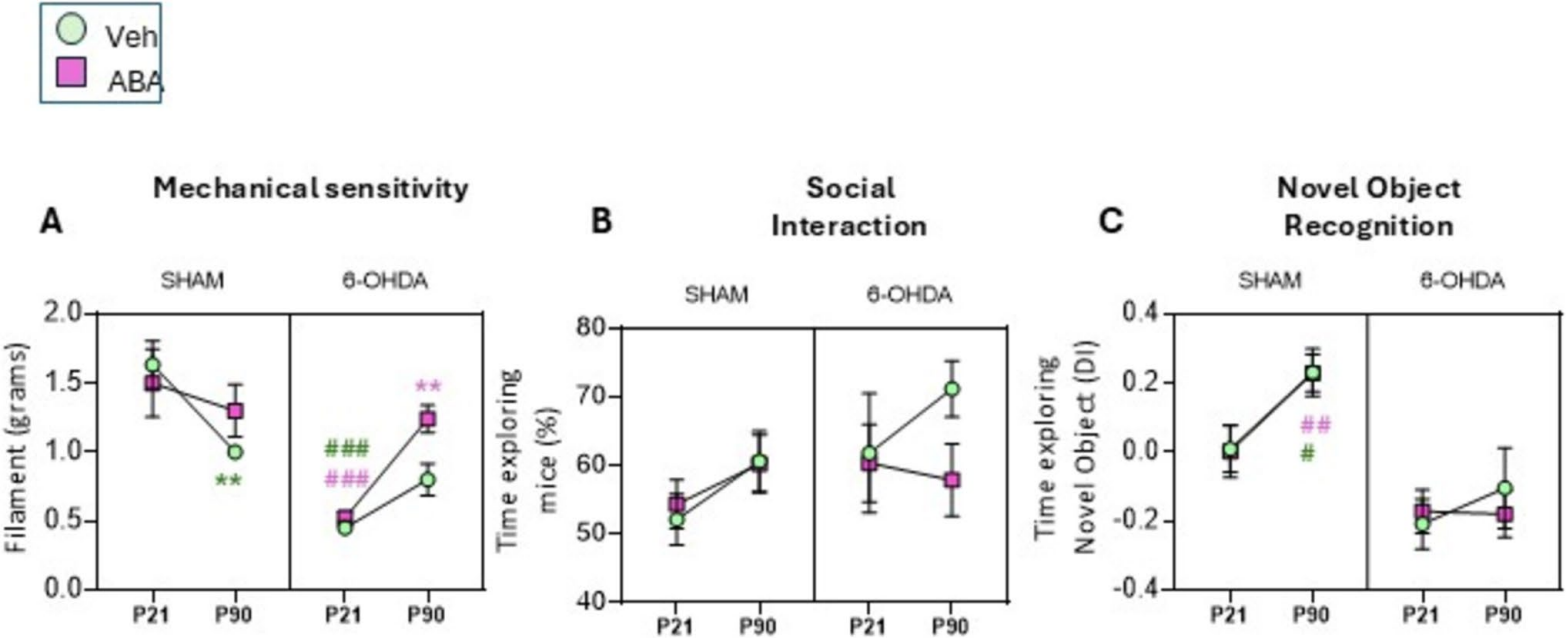
Neonatal 6-OHDA lesion increased mechanical sensitivity and social interaction and impairs recognition memory. Age and ABA treatment alleviates mechanical hypersensitivity in lesioned mice. (**A**) Mechanical threshold (grams of filament) in Von Frey test. (**B**) Time exploring the co-specific mice expressed as the percentage of total time exploring. (**C**) Time exploring (d-index) the novel object in the novel object recognition test. Data are expressed as a DI ((Time exploring novel – time exploring familiar)/total time exploring), presented as mean ± SEM (*n* = 4–11 per condition) and analyzed by Three way-ANOVA with repeated measures, followed by post hoc multiple comparisons test. **p < 0.01 **p21 vs p90**; #*p* < 0.05; ##*p* < 0.01 **sham vs lesion** at indicated age. (pink ABA, green VEH)

**Fig. 4:**
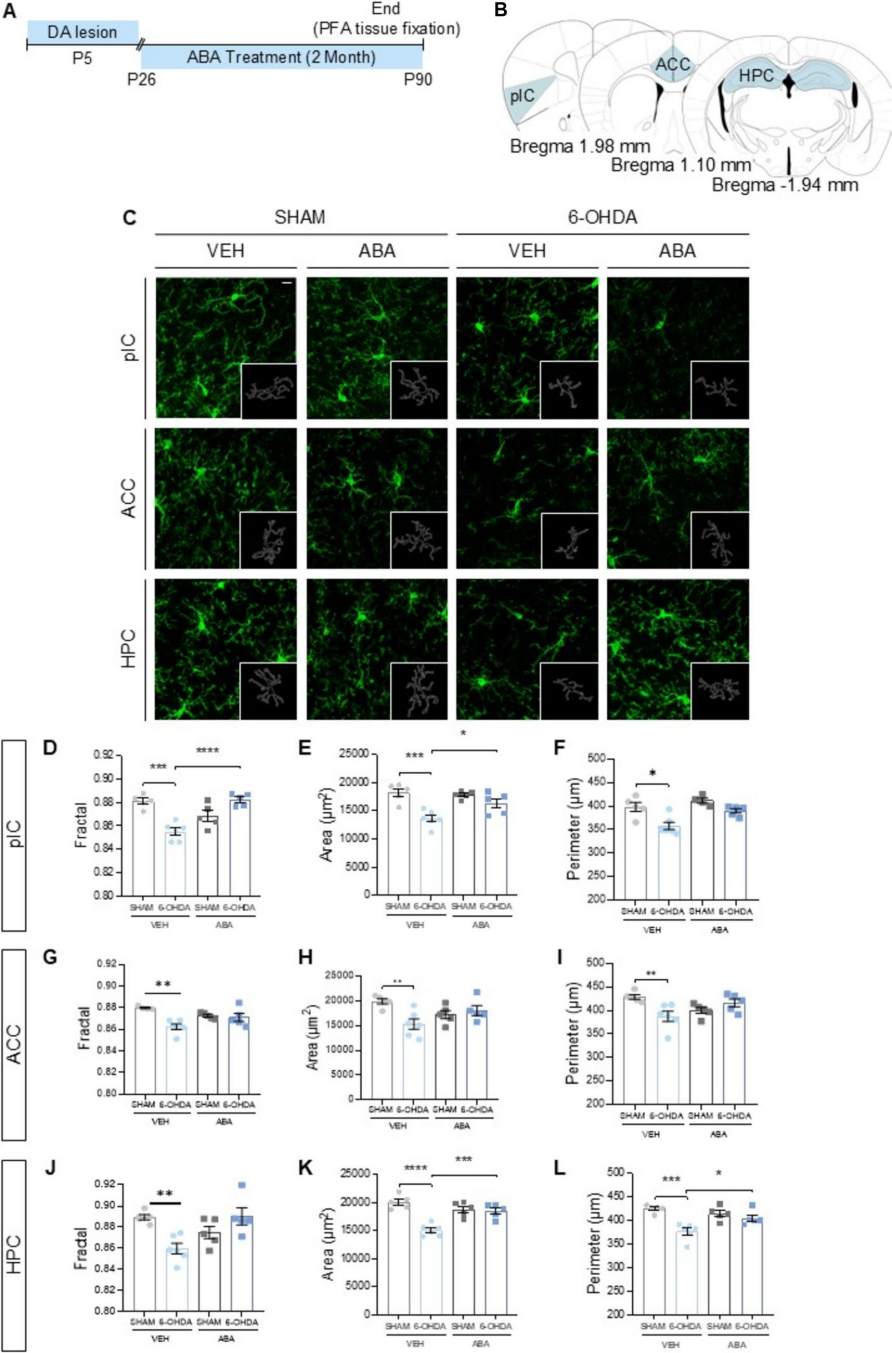
Dopaminergic lesion activates microglia and ABA treatment prevents activation. (**A**) Schematic experimental design for dopaminergic lesion (6-OHDA) indicating the extension of ABA treatment and the moment of brain sample collection to evaluate the neuroinflammation through microglia morphology. (**B**) Schematic representation of the different area that were studied. (**C**) Representative confocal microscopy images from pIC, ACC, and Hippocampus showing Iba1 marker. Inserts in every image are an outline of the microglia cell in the corresponding image. Calibration bar; 10 µm. (**D**) Fractal (**E**) area (µm^2^) and (**F**) perimeter (µm) in pIC. (**G**) Fractal (**H**) area (µm^2^) and (**I**) perimeter (µm) in ACC. (**J**) Fractal (**K**) area (µm.^2^) and **(L)** perimeter (µm) in Hippocampus. Data are expressed as mean ± SEM (*n* = 4–6 per condition, with 5 cells analyzed per animal) and analyzed by Two-way ANOVA followed by post hoc multiple comparisons test (*p < 0.05, **p < 0.01, ***p < 0.001, ****p < 0.0001) and unpaired one-tailed Student t-test (#*p* < 0.05)

**Fig. 5:**
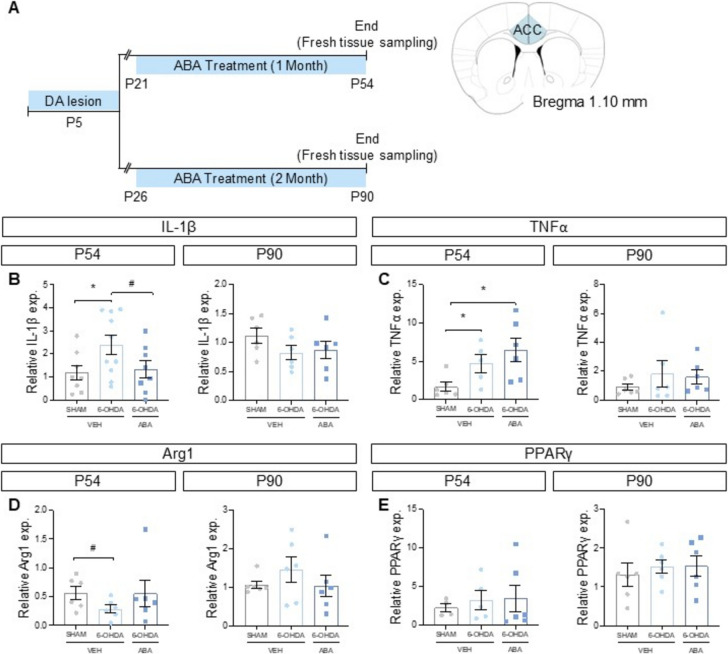
Neonatal 6-OHDA lesion increase pro-inflammatory cytokines expression and decrease anti-inflammatory Arg1 expression. (**A**) Schematic experimental design for dopaminergic lesion (6-OHDA) indicating the extension of ABA treatment and the moment of brain sample collection to evaluate pro-inflammatory and ant-inflammatory cytokines expression. Schematic representation of the area that was studied. (**B**) IL-1β, (**C**) TNFα, (**D**) Arg1 and (**E**) PPARγ expression at P54 (1 month of ABA treatment) and P90 (2 months of ABA treatment). Data are presented as mean ± SEM (*n* = 4–10 per condition) and analyzed using two-tailed Student t-test (*p < 0.05) and one-tailed Student t-test (#*p* < 0.05)

**Fig. 6:**
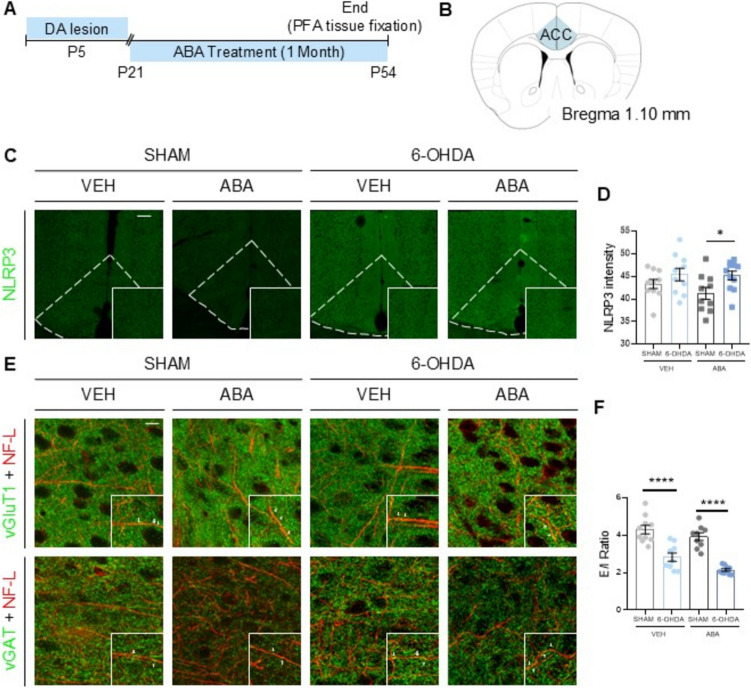
Dopaminergic lesion impairs NLRP3 and E/I ratio. (**A**) Schematic experimental design for dopaminergic lesion (6-OHDA) indicating the extension of ABA treatment and the moment of brain sample collection to evaluate the neuroinflammation and E/I ratio. (**B**) Schematic representation of the area that was studied. (**C**) Representative confocal microscopy images from ACC showing NLRP3 marker. Calibration bar; 100 µm. (**D**) NLRP3 intensity (mean gray value). (**E**) Representative confocal microscopy images from ACC showing vGluT1, vGAT and NF-L markers. Inserts in every image show vGluT1 and vGAT in pre-synaptic terminal. Calibration bar; 100 µm. (**F**) E/I ratio in ACC females. (**G**) vGAT1 quantification (puncta/mm^2^). (**H**) vGLUT1 quantification (puncta/mm.^2^). Data are presented as mean ± SEM (*n* = 9–11 per condition) and analyzed by Two-way ANOVA followed by post-hoc multiple comparisons test (**p < 0.01, ***p < 0.001, ****p < 0.0001)

The analysis was carried out using GraphPad Prism V8 software (GraphPad, La Jolla, CA, USA). Data were subjected to the Shapiro–Wilk test for Gaussian distribution. If normality was confirmed, data were reported as mean ± SEM, with "n" representing the number of independent subjects. A three-way ANOVA with repeated measures was applied to the data in Figs. 2 and 3, followed by post hoc multiple comparison tests. A two-way ANOVA was applied to the data in Figs. 4, 5 and 6. A one-tailed unpaired Student’s t-test was used to analyze the data in Fig. 5. In all cases, the probability threshold was set at α < 0,05.

## Results

As described previously (Meseguer-Beltrán et al. 2023), animals with less than 45% of the VTA area lesioned following 6-OHDA injection were excluded from the analysis.

### Neonatal 6-OHDA Lesions Induced Hyperactivity and Impulsivity in Juvenile Mice. Brain Maturation Reduced Impulsivity, and ABA Treatment Normalized Locomotor Behavior

Spontaneous locomotor activity was assessed by measuring distance traveled and speed during a 10-min open field test. Representative tracking of mice activity is shown (Fig. 2A). To understand the effects of neonatal 6-OHDA lesions, ABA treatment, and time (brain maturation), we conducted a three-way ANOVA with repeated measures to compare behavior at juvenile and adult time points.

The analysis of distance (Fig. 2B) revealed significant overall impacts of time [F(1,38) = 4.871, *p* = 0.0334*] and 6-OHDA [F(1,38) = 19.15, *p* < 0.0001***]. Similarly, for velocity (Fig. 2C), significant effects were observed for time [F(1,39) = 7.341, *p* = 0.01**] and 6-OHDA [F(1,39) = 21.11, *p* < 0.0001***].

Locomotor activity was also influenced by the interaction between the three variables [time × 6-OHDA × ABA], with an interaction effect both in distance [F(1,39) = 6.906, *p* = 0.0122*] and velocity [F(1,39) = 7.814, *p* = 0.008**]. Post hoc Sidak´s multiple comparisons test indicated a significant effect of 6-OHDA in juvenile mice [P21 SHAM-VEH vs. P21 6-OHDA-VEH], with distance showing *p* = 0.0367* and velocity showing *p* = 0.0315*. In adults, significant differences were observed [P90 SHAM-VEH vs. P90 6-OHDA-VEH], with distance showing *p* = 0.0067** and velocity showing *p* = 0.007**.

Lesioned mice remained hyperactive into adulthood (with no difference between P21 and P90), but ABA treatment counteracted the lesion effects [P21 6-OHDA-ABA vs. P90 6-OHDA-ABA], with distance showing *p* = 0.0053** and velocity showing *p* = 0.0017**.

These findings suggest that ABA treatment can help regulate locomotor activity by reducing lesion-induced hyperactivity and potentially preventing the decrease in the spontaneous locomotor activity associated with physiological development.

We next analyzed the effects of the neonatal dopaminergic lesion on EPM performance (Fig. 2D). This paradigm is validated for measuring impulsivity in ADHD (Cho et al. 2014) and aging mice models (Wang et al. 2023), as time spent in the open arms is considered an indicator of risk-taking behavior. Impulsivity was significantly influenced by brain maturation (F (1, 36) = 29.81, *p* < 0.0001****) and dopaminergic lesion (F (1, 36) = 8.051, *p* = 0.0074**).

These results suggest that risk-taking behavior, as measured by the time spent in the open arms of the EPM, is higher in juvenile mice compared to adults, and it is higher in lesioned mice. Brain maturation has a significant impact reducing time in the open arms both in sham and lesioned mice.

### Brain Development Decrease Anxiety and ABA Treatment Exerts an Anxiolytic Effect

Shortened time in the center and increased latency to first crossing into the center area of the open field are indicators of elevated anxiety. A three-way ANOVA analysis revealed that brain maturation (F(1, 36) = 18.59; *p* = 0.0001***) and ABA treatment (F(1, 36) = 4.324; *p* = 0.0448*) significantly increased the time spent in the center of the open field (Fig. 2E). Notably, the neonatal dopaminergic lesion did not show an overall significant effect.

This observation suggests that brain maturation reduces both anxiety and hyperactivity. Multiple comparisons with Fisher correction revealed that ABA treatment synergizes with brain maturation in increasing time spent in the center for lesioned mice [P90 6-OHDA-VEH vs P90 6-OHDA-ABA] *p* = 0.0423*), indicating a potential anxiolytic effect.

The latency to first crossing into the center (Fig. 2F) serves as another anxiety measure in mice. A three-way ANOVA analysis revealed that brain maturation was the primary factor influencing this parameter [F (1, 34) = 5.94, *p* = 0.0202*]. Neither the dopaminergic lesion, nor ABA treatment showed statistically significant impact on this metric.

These comprehensive results strongly suggest that natural brain development progressively downregulates anxious responses and that ABA treatment may offer an anxiolytic effect, as demonstrated in subjects with dopaminergic signaling deficits. The findings highlight the complex interplay between neurological development, pharmacological intervention, and behavioral manifestations of anxiety in neurodevelopmental disorder models.

### Neonatal Dopaminergic Lesion Increased Mechanical Sensitivity, While Age and ABA Treatment Alleviated Mechanical Hypersensitivity in Lesioned Mice

Mechanical sensitivity threshold was assessed using the Von Frey test (Fig. 3A). A three-way ANOVA with repeated measures analyzed the effect of time (brain maturation), 6-OHDA, and ABA treatment. 6-OHDA lesion emerged as the sole factor with a statistically significant overall effect [F (1,17) = 19.15; *p* = 0.0004***]. Brain maturation showed significant interactions with both ABA treatment [TIME x ABA] F (1,17) = 7.484; *p* = 0.0141*] and dopaminergic lesion [TIME × 6-OHDA] F (1,17) = 42.011; *p* < 0.0001****].

To aid in interpreting these findings, Sidak's multiple comparison tests were applied. Juvenile lesioned females were hypersensitive compared to control mice [P21 SHAM-VEH vs. P21 6-OHDA-VEH] (*p* < 0.0003***). Development decreased the threshold in control mice ([P21 SHAM-VEH vs. P90 SHAM-VEH] *p* = 0.0061**), suggesting that brain maturation can intensify sensitivity to mechanical stimuli in control subjects. However, this reduction was not observed in controls treated with ABA. On the other hand, ABA treatment increased the threshold in lesioned mice ([P21 6-OHDA-ABA vs. P90 6-OHDA-ABA] *p* = 0.0042**), indicating that ABA alleviates hypersensitivity in ADHD model mice.

These findings underscore the complex relationship between neurological development, dopaminergic lesions, and mechanical pain sensitivity, highlighting potential therapeutic implications for managing sensory processing in neurodevelopmental disorders.

Furthermore, the results indicate that dopamine deficits increase mechanical pain sensitivity in juvenile mice compared to age-matched controls. While controls exhibit increased sensitivity with brain maturation, ABA treatment counteracts these effects, suggesting its potential application in alleviating pain sensitivity.

### Social Interaction is Not Significantly Altered by Dopamine Deficit or Brain Maturation

Social interaction (SI) was measured through co-specific mouse exploration (Fig. 3B). A three-way ANOVA analysis found no significant effects of development, 6-OHDA, or ABA treatment.

### Neonatal 6-OHDA Lesions Impair Recognition and Spatial Memory in Adult Mice, and ABA Treatment Does Not Rescue this Effect

Next, we aimed to determine whether neonatal 6-OHDA lesions affect novel recognition memory in juvenile and adult female mice using the NOR test (Fig. 3C). For this parameter, dopaminergic deficits [6-OHDA (F(1,33) = 31.84; *p* < 0.0001****] and brain maturation [F(1,33) = 5.978; *p* = 0.05*] had a significant overall impact. Control juvenile mice performed poorly in the test [P21 SHAM (d-index = 0.009 ± 0.068)], but brain maturation improved novel recognition performance [P90 SHAM (d-index = 0.229 ± 0.054)]. However, neonatal lesions prevented this improvement with brain maturation [P21 6-OHDA d-index = −0.20 ± 0.073 and P90 6-OHDA d-index = −0.104 ± 0.117]. ABA treatment did not rescue the lesion-induced impairment.

These results demonstrate that normal development improves performance in the novel recognition task, but this improvement is impaired in mice with neonatal dopaminergic-lesioned mice and cannot be rescued by ABA treatment.

Spatial memory was further analyzed in adult mice using the T-Maze (Supplementary Fig. S1A). Juvenile mice were not tested in this paradigm, as they are too young to perform the task, as confirmed by the NOR test. In adult mice, neonatal lesions reduced the number of entries into the novel arm compared to sham females, as indicated by two-way ANOVA. Lesion had a significant impact [F (1,26) = 16.28 *p* = 0.0004***. Sidak´s multiple comparisons test revealed significant differences between [SHAM-VEH vs. 6-OHDA-VEH] (Supplementary Fig. S1B; *p* = 0.0064**). While ABA treatment could not fully recover this deficit, [SHAM- ABA vs. 6-OHDA-ABA] also showed significant differences (Supplementary Fig. S1B; *p* = 0.0467*).

These findings suggest that neonatal dopamine deficits impair T-maze performance in adult mice, indicating either impaired spatial memory or reduced novelty recognition, and ABA treatment does not rescue this impairment.

### Neonatal 6-OHDA Lesion Promotes Microglia Polarization to a Proinflammatory State in the pIC, ACC, and Hippocampus, while Two Months of ABA Treatment Restore Microglia Morphology

Microglia in three-month-old mice were visualized in several brain regions: the pIC, ACC, and the dentate gyrus of the hippocampus (Fig. 4A-C) using Iba-1 staining. Morphological changes in microglia were assessed based on fractal, area, and perimeter measurements.

In the pIC, analysis of microglia fractal dimension (Fig. 4D) using a two-way ANOVAs followed by Tukey’s multiple comparison test revealed a significant overall lesion effect [F (1, 17) = 22,32, *p* = 0.028*] and interaction, [F (1, 17) = 5,721, *p* = 0.0002***]. Post hoc analysis showed significant differences between control and lesioned mice ([SHAM-VEH vs. 6-OHDA-VEH] *p* = 0.0001***) and between untreated and ABA-treated lesioned mice ([6-OHDA-VEH vs. 6-OHDA-ABA] *p* < 0.0001****).

For microglia area in the pIC (Fig. 4E), the neonatal 6-OHDA lesion had a significant impact [F (1, 17) = 22.32, *p* = 0.0002***]. Tukey’s test identified specific differences between untreated control and lesioned mice ([SHAM-VEH vs. 6-OHDA-VEH] *p* = 0.0004***) and between lesioned mice with and without ABA treatment ([6-OHDA-VEH vs. 6-OHDA-ABA] *p* = 0.036*).

Finally, in the pIC, analysis of microglia perimeter (Fig. 4F) showed significant effects of the lesion [F (1, 17) = 11.33, *p* = 0.0037**] and ABA treatment [F (1, 17) = 8.43, *p* = 0.0099**]. Post hoc tests indicated specific differences between [SHAM-VEH vs. 6-OHDA-VEH] *p* = 0.0353*.

In the ACC, the fractal dimension was significantly affected by the lesion [F (1, 16) = 11.41, *p* = 0.0038**]. While ABA treatment did not show a significant overall effect, it strongly interacted with the lesion [F (1, 16) = 7.926, *p* = 0.0124*]. Tukey’s multiple comparisons test indicated that the lesion reduced microglial ramification ([SHAM-VEH vs. 6-OHDA-VEH] *p* = 0.0036**), but this effect was not observed in the ABA-treated group (Fig. 4G).

For microglial area (Fig. 4H), the lesion interacted significantly with ABA treatment [F (1, 17) = 8.926, *p* = 0.0083**]. Tukey’s tests revealed significant differences between untreated control and lesioned mice [SHAM-VEH vs. 6-OHDA-VEH] (*p* = 0.0095**). analysis of microglial perimeter in the ACC (Fig. 4I) showed a significant interaction [F (1, 17) = 9.272, *p* = 0.0073**], with differences between control and lesioned mice [SHAM-VEH vs. 6-OHDA-VEH] (*p* = 0.026*).

In the hippocampus, fractal dimension (Fig. 4J) was significantly affected by the lesion [F (1, 17) = 7.048, *p* = 0.0167*] and showed an interaction with ABA treatment [F (1, 17) = 5.348, *p* = 0.0335*]. Tukey’s post hoc test indicated that the lesion reduced fractal dimension in untreated mice [SHAM-VEH vs. 6-OHDA-VEH] (*p* = 0.0182**).

For microglial area (Fig. 4K), the 6-OHDA lesion had a significant overall impact [F (1, 17) = 25.38, *p* = 0.0001***] and showed a strong interaction with ABA treatment [F (1, 17) = 21.09, *p* = 0.0003***]. Tukey’s post hoc tests revealed significant differences: [SHAM-VEH vs. 6-OHDA-VEH] (*p* < 0.0001****) and [6-OHDA-VEH vs. 6-OHDA-ABA] (*p* = 0.0008***). Finally, for microglial perimeter in the hippocampus (Fig. 4L), the lesion had a significant overall impact [F (1, 17) = 19.74, *p* = 0.0004***], with a significant interaction observed with ABA treatment [F (1, 17) = 9.117, *p* = 0.0077**]. Post hoc analysis revealed that the lesion significantly reduced microglial perimeter [SHAM-VEH vs. 6-OHDA-VEH] (*p* < 0.0003***), while ABA rescued the lesion-induced reduction [6-OHDA-VEH vs. 6-OHDA-ABA] (*p* = 0.028*).

These data confirm that alterations in microglial morphology induced by neonatal dopaminergic lesions persist into adulthood (3-month-old mice). Furthermore, the results demonstrate that ABA treatment counteracts the effects of the lesion, promoting a more ramified microglial morphology. Importantly, prolonged ABA exposure effectively rescues microglial morphology in the lesioned hippocampus, whereas one month of treatment was insufficient for recovery (Meseguer-Beltrán et al. 2023).

### Dopaminergic Lesions Increase M1 and Decrease M2 Cytokines in Young Mice, and ABA Counteracts this Effect

To further investigate the mechanisms by which dopaminergic lesions and ABA treatment influence behavior, we analyzed molecular alterations in the ACC of two- and three- month-old mice (Fig. 5A).

Pro-inflammatory cytokines IL-1β and TNFα were significantly elevated in two-month-old mice that had undergone a neonatal 6-OHDA lesion [P54 SHAM-VEH vs. P54 6-OHDA-VEH]. IL-1β levels showed a significant increase (*p* = 0.0437*; Fig. 5B), as did TNFα levels (*p* = 0.0347*; Fig. 5C). ABA treatment [P54 6-OHDA-VEH vs. P54 6-OHDA-ABA] significantly reduced IL-1β expression (*p* = 0.0422*; Fig. 5B) but had no effect on TNFα levels (Fig. 5C).

Conversely, Arg1, a marker of M2 microglia, was significantly reduced in two- month-old lesioned females compared to age-matched controls [P54 SHAM-VEH vs. P54 6-OHDA-VEH] (*p* = 0.0296 *; Fig. 5D). Notably, these lesion-induced differences were not observed in three-month-old mice [P90 SHAM-VEH vs. P90 6-OHDA-VEH], as IL-1β (Fig. 5B), TNFα (Fig. 5C), and Arg1 (Fig. 5D) levels did not differ significantly.

Interestingly, ABA treatment mitigated lesion-induced differences in cytokine profiles, suggesting that ABA prevents the pro-inflammatory (M1) microglial phenotype in two-month-old lesioned mice.

Finally, since ABA is known to increase PPARγ expression in vivo (Bassaganya-Riera et al. 2011), we evaluated PPARγ levels in our study. However, we observed no significant alterations under any condition (Fig. 5E).

These results suggest that, in two-month-old lesioned female mice, microglia exhibit a pronounced pro-inflammatory (M1) status, characterized by elevated IL-1β and TNFα levels and reduced Arg1 expression (a marker of M2 microglia). ABA treatment effectively counteracts this pro-inflammatory state. Although the cytokine profile normalizes with age, the early inflammatory changes induced by dopamine deficit may contribute to long-lasting behavioral alterations.

### NLRP3 Inflammasome Expression is not Significantly Altered by the 6-OHDA Lesion

To further investigate the early cytokines alterations and considering the relationship between dopamine and the NLRP3 inflammasome (Yan et al. 2015) as well as the pathophysiology of NLRP3 inflammasome overactivation (Menu and Vince 2011; Guo et al. 2015); we measured NLRP3 expression in the ACC of young female mice (Fig. 6A-B) using immunofluorescence staining (Fig. 6C).

Quantification of fluorescence intensity, followed by two-way ANOVA analysis (Fig. 6D), revealed a significant effect of the lesion on NLRP3 expression [(F (1, 37) = 9.172, *p* = 0.0045**]. ABA treatment reduced NLRP3 expression only in SHAM mice, not in the 6-OHDA group, with significant differences observed between [SHAM-ABA vs. 6-OHDA-VEH] (*p* = 0.0284*) and [SHAM-ABA vs. 6-OHDA-ABA] (*p* = 0.0346*).

### Neonatal 6-OHDA Lesion Alters the Excitatory/Inhibitory Ratio, while One Month of ABA Treatment Increases vGAT. At the Postsynaptic Level, no Alterations are Observed Under Any Condition

Given the significant role of excitation/inhibition (E/I) ratio imbalance in neurodevelopmental disorders (van Hugte et al. 2023) and the strict regulation of the E/I balance by microglia (Sood et al. 2021), we hypothesized that microglial alterations in this model could disrupt the E/I balance and that ABA treatment might rescue this disruption. To test this hypothesis, we analyzed the expression of vesicular glutamate transporter 1 (vGluT1) and vesicular GABA transporter (vGAT), markers associated with the E/I ratio (Fattorini et al. 2017). Immunodetection of vGluT1 and vGAT was performed in conjunction with the axonal marker Neurofilament-L in the ACC (Fig. 6E).

Quantification followed by two-way ANOVA analysis (Fig. 6F) revealed significant effects of the neonatal 6-OHDA lesion (F (1, 34) = 72.93, *p* < 0.0001****) and ABA treatment (F (1, 34) = 7.895, *p* = 0.0082**) on the E/I ratio. Tukey´s post hoc tests confirmed that the lesion significantly decreased the E/I ratio compared to controls [SHAM-VEH vs. 6-OHDA-VEH] (*p* < 0.0001****), primarily due to increased vGAT expression (Fig. 6G *p* = 0.093**). Two-way ANOVA revealed no significant differences in vGluT1 expression (Fig. 6H). Notably, one month of ABA treatment further reduced the E/I ratio in lesioned mice [6-OHDA-VEH vs. 6-OHDA-ABA] (*p* < 0.0001****), primarily by increasing vGAT expression (Fig. 6G *p* < 0.001****).

To investigate changes at the postsynaptic level, we evaluated the colocalization of Homer1, a protein that regulates metabotropic glutamate receptor function (Tao-Cheng et al. 2014), and MAP2, a microtubule-associated protein enriched in dendrites (Supplementary Fig. S2A). No significant alterations were observed with either 6-OHDA lesion or ABA administration (Supplementary Fig. S2B).

These findings suggest that neonatal 6-OHDA lesion disrupts the E/I balance, primarily through changes in inhibitory markers. ABA administration increased the inhibitory marker vGAT, correlating with the rescue of microglia morphology and potentially providing a mechanism for ADHD treatment.

## Discussion

Female subjects are significantly underrepresented in preclinical studies of the neonatal dopaminergic lesion model of ADHD. To address this gap, we used female mice to examine the effects of brain maturation and anti-inflammatory treatment on ADHD-related behaviors, comparing behaviors at juvenile (P21) and adult (P90) time points. These stages correspond to approximately 3 years and 20 years of human brain development, respectively (Semple et al. 2013).

Our study demonstrates that neonatal dopaminergic lesions induce long-term effects on behavior. Symptoms such as hyperactivity and hypersensitivity to mechanical stimuli are evident in juvenile mice and persist into adulthood. In contrast, impulsivity decreases with brain maturation and cognitive impairment emerges only in adulthood. Notably, anxiety levels are unaffected by the lesion and naturally decrease with development.

Interestingly, ABA treatment alleviated most symptoms observed in three-month-old mice, except for cognitive impairment. Previous studies, including ours, have highlighted the anti-inflammatory benefits of ABA in animal models, including metabolic syndrome (Sánchez-Sarasúa et al. 2016) and Alzheimer´s disease (Espinosa-Fernández et al. 2019). Both our research (Meseguer-Beltrán et al. 2023) and other studies (Corona 2020) suggest a strong link between neuroinflammation and hyperactivity and hypersensitivity in ADHD models.

ABA was therefore investigated as a potential chronic therapeutic treatment. Dopaminergic lesions induced hyperactivity and impulsive behavior in young female mice, consistent with findings from male ADHD models (Ueno et al. 2002; Mortimer et al. 2019; Bouchatta et al 2018). In contrast, control mice exhibited reductions in spontaneous locomotor activity and impulsivity with maturation, reflecting developmental changes in prefrontal cortical structures observed in humans (Romer 2010). Among lesioned females, impulsivity decreased with brain maturation but remained higher than that of age-matched controls, while hyperactivity persisted. These enduring traits parallels behaviors observed in some ADHD patients, where impulsivity into adulthood is associated with increased risks such as substance abuse and automobile accidents (Kosheleff et al. 2023).

Two-months of ABA treatment effectively reduced hyperactivity only in lesioned female mice, consistent with our previous findings (Meseguer-Beltrán et al. 2023), and other studies. For example, ABA has been shown to rescue motor deficits in Parkinson’s disease (Shabani et al. 2023) and in a rodent model of harmaline-induced motor disabilities (Shabani and Naderi 2022). Hyperactivity and impulsivity are strongly influenced by dopamine deficiency and/or insufficient activation of the dopamine D1 receptor in the ACC (Puig et al. 2014; Yates et al. 2016; Rahi and Kumar 2021).

Interestingly, ABA treatment did not alter dopaminergic signaling in the VTA (Meseguer-Beltrán et al. 2023), strengthening the hypothesis that ABA’s anti-inflammatory effects may specifically counteract hyperactivity caused by dopaminergic deficits. Additionally, ABA counteracted the age-related decline in activity observed in control mice, suggesting that ABA might have revitalizing effects on healthy individuals.

Anxiety in rodents is often assessed using the open field paradigm, where increased time spent in the center and reduced latency to the first crossing are typically indicative of lower anxiety. Also, the EPM is commonly used to evaluate anxiety, whereas impulsivity and attention are typically evaluated through the 5 -choice serial reaction time task. However, the EPM has also been successfully validated for assessing impulsive behavior in ADHD rat model (Cho et al. 2014) and in an aging model (Wang et al. 2023), considering that spending time in the open arms can be interpreted as risk-taking behavior.

Measuring anxiety using the open field can be particularly challenging in a hyperactivity model, as increased center time and reduced latency might also result from hyperactivity or impulsivity rather than reflecting true anxiety levels.

Our longitudinal experimental design provides a more comprehensive perspective for understanding these factors. Age (brain maturation) decreased locomotor activity in control mice but not in lesioned mice. Similarly, age increased the time spent in the center of the open field (indicating reduced anxiety) in control, but not in lesioned mice, suggesting that the neonatal lesion counteracts brain maturing effect, rendering potentially more anxious adults. Age also reduced the time in the open arms of the EPM (indicating reduced impulsivity) in both control and lesioned mice. However, in the lesioned group, impulsive behavior in adulthood did not reach the lower levels observed in control. These results parallel human development, where adults with ADHD often exhibit persistent hyperactive and impulsive behavior.

Interestingly, ABA treatment in lesioned mice decreased hyperactivity and increased the time spent in the center of the open field (indicating reduced anxiety) in adults. This suggests that ABA has anxiolytic effects and, importantly, helps regulate hyperactivity in a dopamine-deficient model.

Our data support previous studies demonstrating that ABA exerts anxiolytic effects by modulating Protein Kinase C (Naderi et al. 2017), and ERK signaling (Naderi et al. 2019). This may be explained by the role of neuroinflammation in contributing to anxiety and depressive behavior (Guo et al. 2023). Furthermore, neuroinflammation in the ACC is strongly associated to mood disorders (Matisz and Gruber 2022; Zhou et al. 2022), while activity in the posterior insular cortex correlates with heart-induced anxiety (Couderc and Beyeler 2023; Hsueh et al. 2023). These findings are particularly relevant for ADHD management, as anxiety is a comorbid symptom in approximately 50% of cases (Bishop et al. 2019). This underscores the potential of targeting neuroinflammation as an alternative therapeutic approach for treating both ADHD and its associated anxiety symptoms.

Another symptom associated with ADHD is hypersensitivity to various stimuli, which is more common in girls (Wolff et al. 2016) and women (Asztély et al. 2019). Our study found that neonatal dopaminergic lesions induced mechanical hypersensitivity in juvenile females compared to age-matched controls, consistent with previous research (Bouchatta et al. 2022; Meseguer-Beltrán et al. 2023). Interestingly, sensitivity to mechanical stimuli increased with normal development in sham untreated mice, mirroring observations in healthy humans (El Tumi et al. 2017). In contrast, brain maturation mitigated hypersensitivity in lesioned mice.

Notably, ABA treatment reduced sensitivity to mechanical stimuli in both lesioned and control mice, counteracting the effects of both the neonatal dopamine deficit in the lesioned group as well as the age-related increase in sensitivity observed in controls. This finding is supported by recent studies showing that ABA alleviates neuropathic pain by reducing inflammation in the spinal cord (Maixner et al. 2022). Since neuroinflammation is closely linked to pain (Vergne-Salle and Bertin 2021), we hypothesize that ABA’s reduction of neuroinflammation in the ACC underlies its effect on ameliorating hypersensitivity.

Hyper-sociability has been reported in males of the spontaneous hypertensive model of ADHD (Baek et al. 2014). Our study found that sociability increased with development, and in agreement with the hypertensive model, dopaminergic lesion exacerbated sociability in adults. While social difficulties are commonly reported in ADHD patients (Carpenter Rich et al. 2009), drawing a direct comparison to human behavior remains complex. Further research is needed to explore the relationship between dopamine deficits and the dysregulation of striatal neuropeptides involved in sociability, such as arginine, vasopressin, and oxytocin, which are critical for social behavior (Cataldo et al. 2018; Ghirardi et al. 2018).

Another possibility is that increased impulsivity and hyperactivity led to more time spent exploring co-specific mice. Supporting this hypothesis, ABA treatment reduced hyper-sociability in lesioned females, likely due to its effects on regulating locomotor activity.

Cognitive function can be impaired in ADHD patients (van Ewijk et al. 2014; Johnson et al. 2021). Our study confirmed that dopamine deficits impair spatial working memory, as evidenced by deficits in both the NOR and T-maze paradigms. However, ABA treatment did not restore memory function, contrary to our previous findings in other models (Sánchez-Sarasúa et al. 2016; Espinosa-Fernández et al. 2019). This discrepancy may stem from the reliance of these paradigms on novelty recognition, a process heavily dependent on dopamine signaling (Clos et al. 2019; Titulaer et al. 2021). Consequently, while ABA’s anti-inflammatory effects may alleviate some aspects of neuroinflammation, they may not be sufficient to counteract memory impairments in the absence of adequate dopamine levels. Further research is required to explore ABA’s potential therapeutic role on attentional processes using tasks that are less dependent on novelty recognition.

To investigate how ABA exerts its effects, we assessed microglia morphology in the ACC of adult animals. Microglia morphology reflects activation status (Hovens et al. 2014; Fernández-Arjona et al. 2017; Young and Morrison 2018), and our findings revealed that neonatal 6-OHDA lesions led to persistent microglia activation in adults. ABA treatment effectively restored microglia morphology across all studied areas, consistent with previous reports (Espinosa-Fernández et al. 2019; Meseguer-Beltrán et al. 2023). Notably, ABA improved microglial morphology in the hippocampus, although this did not translate to improved performance in memory tests.

In our evaluation of microglial status, we found that, in two-month-old mice, dopamine lesions increased the expression of pro-inflammatory cytokines IL-1β and TNFα mRNA, while Arg1, a marker of M2 microglia, was decreased in the ACC. ABA treatment reduced IL-1β and Arg1 levels but had no significant effect on TNFα mRNA expression. In older mice (P90), these cytokine alterations were no longer observed, despite the persistence of microglial and behavioral abnormalities.

These findings suggest that early inflammatory markers can induce long-lasting behavioral changes, as has been observed in other developmental disorders (Catale et al. 2020). Our data indicate that early intervention targeting inflammation with ABA holds promise as a therapeutic approach to alleviate adult behavioral symptoms.

To further elucidate the mechanism of ABA´s action, we measured PPARγ mRNA expression in the brains of two-month-old female mice. ABA´s effects are mediated by its direct binding to Lanthionine Synthetase Component C-like Protein 2 (LANCL-2) (Sturla et al. 2011; Possemato et al. 2023), which can activate PPARγ function (Kooshki et al. 2021). Both mechanisms are strongly associated with the regulation of inflammation (Bassaganya-Riera et al. 2011). Contrary to earlier findings in the spleen (Bassaganya-Riera et al. 2011), we found that ABA did not increase PPARγ expression in the brain. This discrepancy may be due to differences in the timing, or the specific tissue examined. While our findings do not rule out PPARγ activation, further research is needed to clarify its role in ABA's effects on ADHD symptoms.

The NLRP3 inflammasome mediates the maturation of IL-1β to its active form (Kelley et al. 2019). Dopamine deficits have been shown to activate NLRP3 in primary human microglia and a mouse model of Parkinson’s disease (Pike et al. 2022). We hypothesized that dopamine deficits would increase NLRP3 expression in the ACC, correlating with observed microglial morphological changes, but this effect was not observed. Since we measured NLRP3 protein levels and IL-1β mRNA expression, we cannot rule out altered NLRP3 function. Further experiments evaluating active cytokines are needed to clarify NLRP3's role in ADHD models with dopamine deficits.

Beyond their inflammatory properties, microglia play a key role in regulating the excitation/inhibition (E/I) ratio (Fan et al. 2023). An imbalance in the E/I ratio is a fundamental factor underlying various neuropsychiatric and neurodevelopmental disorders (LeBlanc and Fagiolini 2011). Specifically, E/I imbalances in the ACC and insula (Bai et al. 2019) are associated with heightened nociception in animal models of neurodevelopmental disorders (Qi et al. 2022). In our model, we observed an E/I ratio imbalance; however, unlike other studies that report increased hyperexcitability (Bouchatta et al. 2022), we found reduced vGluT1 and increased vGATs, suggesting hypoexcitability.

Interestingly, microglia can increase glutamate release under inflammatory conditions (Takeuchi et al. 2005; Takeuchi 2013a, b), potentially leading to excitotoxicity regardless of the presynaptic neuron vGluT1 expression. Furthermore, dopaminergic dysfunction can impair EAAT channel activity in astrocytes and neurons, reducing glutamate reuptake from the synapse (Rose et al. 2018).

Remarkably, we found that ABA treatment increased vGAT expression without altering vGluT1. This finding aligns with studies showing that stimulating GABAergic tone in the ACC of a rat model of chronic inflammatory pain alleviates both pain and pain-induced anxiety (Yamada et al. 2012; Shao et al. 2021). Other reports have demonstrated that ABA can facilitate GABA_A_​ function via PPARγ activation (Madadzadeh et al. 2021). Further studies using calcium imaging and electrophysiology are required to confirm ABA's role in neuronal excitability.

In summary, our results suggest that dopamine deficit-induced neuroinflammation triggers hyperactivity, risk-taking behavior, hypersociability, and hypersensitivity. Notably, brain development significantly ameliorates the severity of some symptoms, such as impulsivity, and normalizes the cytokine profile but does not restore microglial morphology. ABA alleviated hyperactivity, hypersensitivity, and increased sociability in adult females, concurrent with improvements in microglial morphology and the rescue of IL-1β and Arg1 expression at early stages. Additionally, ABA may influence the E/I ratio by increasing vGAT levels, likely through the regulation of microglial activity.

These results contribute to the growing body of evidence supporting ABA as a potential anti-inflammatory molecule. Unlike other phytohormones, ABA is also a human hormone (for review see Gharib et al. 2024).

## Conclusions

ADHD has traditionally been associated with dopaminergic alterations (Solanto; Avale et al. 2004). However, our findings suggest that core symptoms might stem from microglia overactivation, triggered by dopamine deficits in this model, but also by other mechanisms under other conditions. This insight highlights the complex etiology of ADHD, supporting evidence linking the disorder to inflammatory conditions such as allergic or atopic diseases (Schans et al. 2017; Miyazaki et al. 2017). Furthermore, it offers a potential explanation for why some patients fail to respond to current treatments like methylphenidate or atomoxetine.

Our study provides a plausible rationale for the diverse presentations of the disorder, whether rooted in genetic dopamine dysfunction or environmental inflammatory factors. This underscores the importance of effective patient stratification to tailor treatments appropriately and minimize adverse effects. Additionally, our findings demonstrate the beneficial effects of ABA in regulating behaviors associated with improved microglial function, as well as upregulated vGAT expression. This novel discovery holds significant potential for advancing treatment strategies in neurological and psychiatric disorders.

## Supplementary Information

Below is the link to the electronic supplementary material.

Supplementary file1 (DOCX 289 KB)

Supplementary file2 (DOCX 15 KB)

## Data Availability

All data supporting the findings of this study are available within the paper and its Supplementary Information.
