# Supplementary material for: Abscisic Acid Rescues Behavior in Adult Female Mice in Attention Deficit Disorder with Hyperactivity Model of Dopamine Depletion by Regulating Microglia and Increasing Vesicular GABA Transporter Expression
Source: J Neuroimmune Pharmacol. 2025 Apr 16;20(1):39. doi: 10.1007/s11481-025-10186-6 (PMC12000189; doi:10.1007/s11481-025-10186-6)
Supplement: Supplementary file 1 — Supplementary file1 (DOCX 289 KB) [file 11481_2025_10186_MOESM1_ESM.docx]

**Abscisic Acid rescues behavior in adult female mice in Attention Deficit Disorder with Hyperactivity model of dopamine depletion by regulating microglia and increasing Vesicular GABA Transporter expression.**

Maria Meseguer-Beltrán^1,2^; Sandra Sánchez-Sarasúa^1,2,3^; Nóra Kerekes^4^, Marc Landry^3^; Matías Real-López^2,5^; Ana María Sánchez-Pérez^1,2,*^

*^1^Institute of Advanced Materials (INAM), Universitat Jaume I, Castellón, Spain.*

*^2^Faculty of Health Sciences, Universitat Jaume I, Castellón, Spain.*

*^3^CNRS UMR 5293, Institut des Maladies Neurodégénératives, Centre Paul Broca-Nouvelle Aquitaine, University of Bordeaux, Bordeaux, France.*

*^4^Department of Health Sciences, University West, 46186 Trollhättan, Sweden.*

*^5^Serious Mental Disorder Program in Childhood and Adolescence, Provincial Hospital Consortium of Castellón, Castellón, Spain.*

* To whom correspondence may be addressed: Ana María Sánchez-Pérez ([sanchean@uji.es](mailto:sanchean@uji.es))

**Acknowledgments:** We would like to thank *Servicios de Experimentación Animal (SEA)* of University Jaume I for the maintenance and care of the experimental animals and S*ervicios Centrales* (Confocal microscopy) of University Jaume I for obtaining the images. We also thank and BioRender.com for creating all the figures.

**
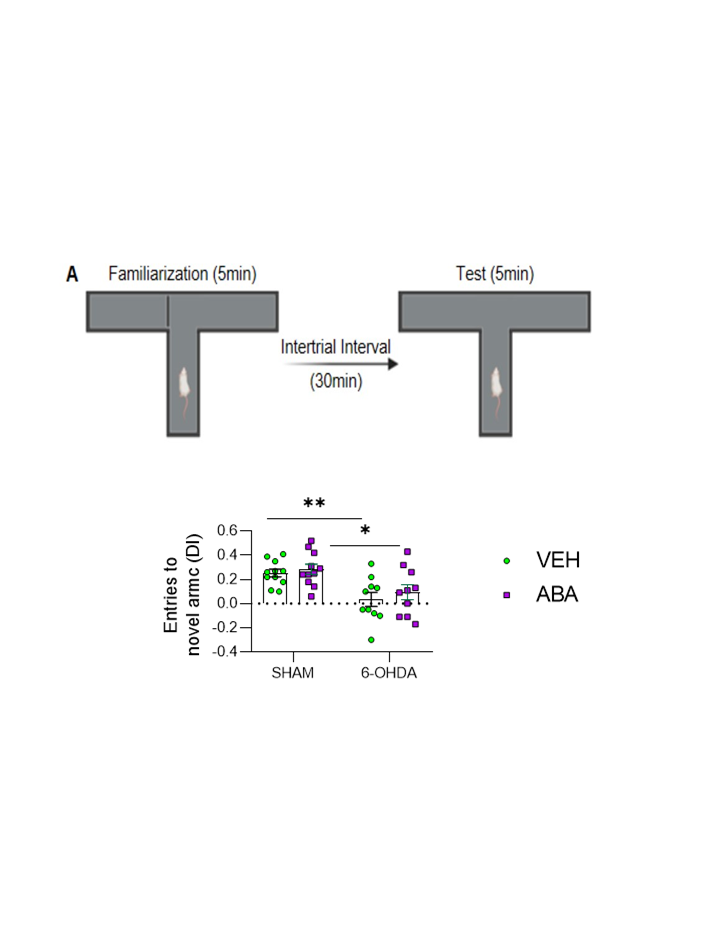
**

**Supplementary Figure S1. Neonatal dopaminergic lesion impairs spatial memory in adults. (A)** Schematic representation of T-maze test. **(B)** Number of entries (d-index) to the novel arm. Data are expressed as a DI ((Time exploring novel – time exploring familiar)/total time exploring), presented as individual points and mean ± SEM (n = 9-10 per condition), analyzed by 2-WAY ANOVA (# p < 0.05, ## p < 0.01).

**
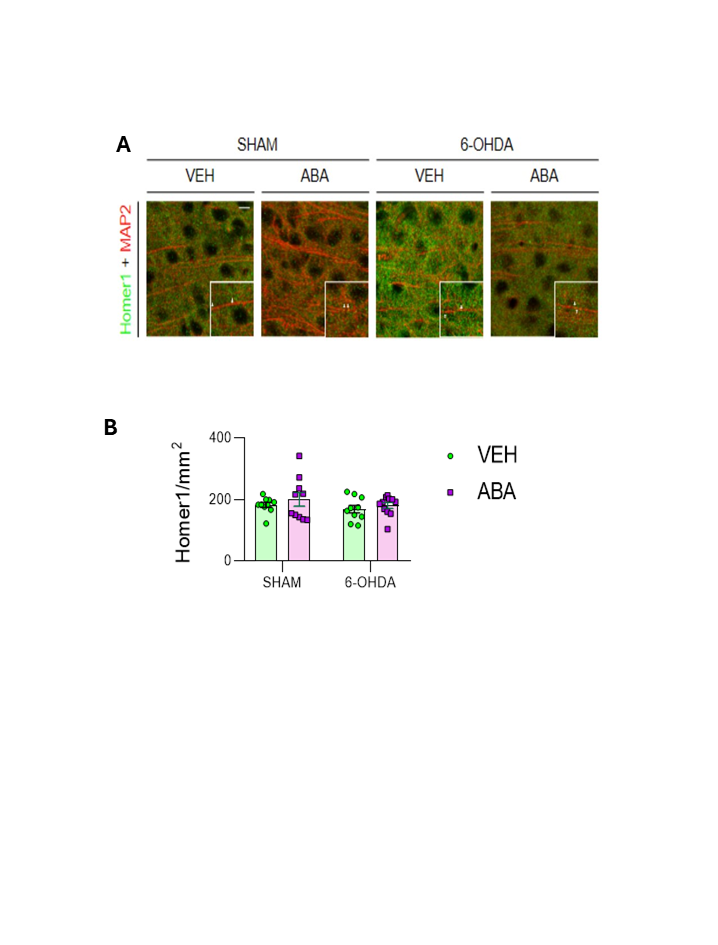
**

**Supplementary Figure S2. Dopaminergic lesion does not affect Homer1 puncta density. (A)** Representative confocal microscopy images from ACC showing Homer1 and MAP2 markers. Inserts in every image show Homer 1 in post-synaptic terminal. Calibration bar; 100 µm. **(B)** vGluT1 **(C)** vGAT and **(D)** Homer1 puncta density (number of puncta/mm2) in ACC females. Data are presented as mean ± SEM (n = 9-10 per condition) and analyzed using two-way ANOVA, followed by post hoc test (* p < 0.05, *** p < 0.001, **** p < 0.0001).
