# Supplementary material for: Abscisic Acid Rescues Behavior in Adult Female Mice in Attention Deficit Disorder with Hyperactivity Model of Dopamine Depletion by Regulating Microglia and Increasing Vesicular GABA Transporter Expression
Source: J Neuroimmune Pharmacol. 2025 Apr 16;20(1):39. doi: 10.1007/s11481-025-10186-6 (PMC12000189; doi:10.1007/s11481-025-10186-6)
Supplement: Supplementary file 2 — Supplementary file2 (DOCX 15 KB) [file 11481_2025_10186_MOESM2_ESM.docx]

**Supplementary Table S1.** List of primers sequences used in qRT-PCR.

| Gene | Accession number | Forward primer (5’->3’) | Reverse primer (5’->3’) |
| --- | --- | --- | --- |
| Arg1 | U51805.1 | AACACGGCAGTGGCTTTAACC | GGTTTTCATGTGGCGCATTC |
| GAPDH | NM_001289726.2 | ACCACAGTCCATGCCATCAC | TCCACCACCCTGTTGCTGTA |
| IL-1β | NM_008361.4 | CCCTGCAGCTGGAGAGTGTGG | TGTGCTCTGCTTGAGAGGTGCT |
| PPARγ | NM_001127330 | CCACCAACTTCGGAATCAGCT | TTTGTGGATCCGGCAGTTAAGA |
| TNF α | NM_013693.3 | AGCCCCCAGTCTGTATCCTT | CTCCCTTTGCAGAACTCAGG |
